# Interventions for increasing ankle joint dorsiflexion: a systematic review and meta-analysis

**DOI:** 10.1186/1757-1146-6-46

**Published:** 2013-11-14

**Authors:** Rebekah Young, Sheree Nix, Aaron Wholohan, Rachael Bradhurst, Lloyd Reed

**Affiliations:** 1 School of Clinical Sciences, Queensland University of Technology, Kelvin Grove, Brisbane QLD 4059, Australia; 2 Institute of Health and Biomedical Innovation, Queensland University of Technology, Kelvin Grove QLD 4059, Australia; 3 Foot Fitness Podiatry Clinic, Grange, QLD 4051, Australia

**Keywords:** Ankle joint, Range of motion, Non-surgical intervention, Stretching, Biomechanics

## Abstract

**Background:**

Ankle joint equinus, or restricted dorsiflexion range of motion (ROM), has been linked to a range of pathologies of relevance to clinical practitioners. This systematic review and meta-analysis investigated the effects of conservative interventions on ankle joint ROM in healthy individuals and athletic populations.

**Methods:**

Keyword searches of Embase, Medline, Cochrane and CINAHL databases were performed with the final search being run in August 2013. Studies were eligible for inclusion if they assessed the effect of a non-surgical intervention on ankle joint dorsiflexion in healthy populations. Studies were quality rated using a standard quality assessment scale. Standardised mean differences (SMDs) and 95% confidence intervals (CIs) were calculated and results were pooled where study methods were homogenous.

**Results:**

Twenty-three studies met eligibility criteria, with a total of 734 study participants. Results suggest that there is some evidence to support the efficacy of static stretching alone (SMDs: range 0.70 to 1.69) and static stretching in combination with ultrasound (SMDs: range 0.91 to 0.95), diathermy (SMD 1.12), diathermy and ice (SMD 1.16), heel raise exercises (SMDs: range 0.70 to 0.77), superficial moist heat (SMDs: range 0.65 to 0.84) and warm up (SMD 0.87) in improving ankle joint dorsiflexion ROM.

**Conclusions:**

Some evidence exists to support the efficacy of stretching alone and stretching in combination with other therapies in increasing ankle joint ROM in healthy individuals. There is a paucity of quality evidence to support the efficacy of other non-surgical interventions, thus further research in this area is warranted.

## Background

Ankle joint equinus occurs when there is reduced dorsiflexion range of motion (ROM) available at the ankle. Studies have shown that the presence of equinus deformity may cause healthy individuals to adopt compensatory gait patterns such as genu recurvatum, early heel lift and excessive subtalar joint pronation [1,2] in addition to altering their biomechanical function in gait. Altered biomechanics may predispose individuals to the development of pathologies such as metatarsalgia, ankle sprain and medial tibial traction periostitis as well as Achilles tendinopathy, plantar fasciopathy and gastrocnemius strain in sporting populations [1-4]. Restricted ROM at the ankle joint has been associated with poor balance and an increase in falls risk in the elderly [5] and furthermore, as equinus increases total plantar pressure acting on the forefoot, it has been linked to a prolongation of the healing time of plantar forefoot ulcers in diabetic patients [6].

There is lack of consensus within the literature regarding the degree of restriction that defines an equinus deformity [7]. Traditionally, less than 10 degrees of dorsiflexion has been cited as an indicator of ankle equinus [8], however, less than zero degrees and less than five degrees are also commonly used markers in biomechanics and sports medicine studies [9,10]. Equinus may result from shortening or contracture of the gastrocnemius or soleus muscles, bony restriction, structural abnormalities of the forefoot, or pathologies causing joint stiffness [7]. Methods of assessment for ankle joint equinus are inconsistent in the literature. Ankle joint ROM has been assessed in weight-bearing and non-weightbearing positions with the knee flexed or extended [11]. Various instruments such as goniometers, inclinometers and dynamometers, as well as different anatomical landmarks have been used to quantify the ROM available [7].

Clinicians screen for ankle joint equinus routinely as part of a lower limb biomechanical assessment and treat equinus conservatively, regardless of ankle joint pathology, to improve biomechanical function of the lower limb [10,11]. A variety of interventions have been proposed to increase actual or functional dorsiflexion ROM at the ankle joint, including stretching, warm up and use of ultrasound [1-6,9,12-32]. Previous systematic reviews have investigated the effects of such interventions in increasing ankle joint dorsiflexion ROM in the context of ankle injuries [33] or neuromuscular disease [34]. However, there has been no synthesis of the literature investigating the efficacy of a range of interventions to increase ankle joint dorsiflexion in otherwise healthy individuals with an incidental finding of ankle equinus. Therefore, the purpose of this systematic review and meta-analysis was to investigate the effects of conservative interventions on increasing ankle joint dorsiflexion range of motion in healthy individuals.

## Methods

### Search strategy and study inclusion

Electronic databases were searched without date or language delimiters (Embase, Medline, Cochrane and CINAHL) using keyword searches, as follows:

(Ankle AND equinus) OR (ankle AND joint AND range AND motion) OR (ankle AND dorsiflex* AND range AND motion) OR (ankle AND rocker)

AND

(dorsiflex* AND lunge AND test) OR treat* OR assess* OR measure* OR interven* OR clinical OR apparatus OR tool OR device OR instrument

The only search parameter applied was the human delimiter. Titles and abstracts were screened for relevance to the research question, and full text evaluations were performed on potentially relevant studies using predetermined criteria. A hand search of the reference lists of all relevant studies was undertaken to identify further eligible studies. Studies were selected for inclusion in this review based on the following criteria:

● Included a sample of healthy, human participants;

● Assessed a conservative (non-surgical) intervention for increasing ankle joint dorsiflexion; and

● Measured and reported passive ankle joint dorsiflexion values before and after intervention.

Studies were excluded based on the following criteria:

● Included participants with spastic equinus, talipes equinovarus or other pathology;

● Included participants with a history of ankle joint injury;

● Assessed surgical interventions.

### Quality assessment

Two authors (RY, AW) independently assessed the included studies against a modified PEDro scale (Table 1) [35]. The two authors met to discuss the PEDro scale rating system prior to undertaking quality assessments in order to ensure clear understanding of assessment criteria. A consensus meeting resolved disagreements between assessors and a third party was available to provide mediation if required. Consensus on all criteria was reached without need for third party mediation.

**Table 1:** Results from quality assessment (23 studies)

**Study ID**	**Ref**	**1**	**2**	**3**	**4**	**5**	**6**	**7**	**8**	**9**	**10**	**11**	**12**	**13**	**14**	**Quality score (/14)**
Bohannon 1994	[31]	-	+	-	+	-	-	+	+	+	+	+	-	+	-	8
Christiansen 2008	[12]	+	+	+	+	-	-	+	+	+	+	+	+	+	-	11
Dananberg 2000	[13]	+	-	-	-	-	+	-	+	-	-	+	-	-	-	4
De Souza 2008	[39]	+	-	-	-	-	-	+	+	+	+	+	+	+	-	8
Dinh 2011	[1]	+	+	+	+	-	+	-	-	+	+	+	+	+	-	10
Draper 1998	[14]	-	+	-	-	-	-	-	-	-	+	+	-	+	-	4
Etnyre 1986	[15]	-	+	-	-	-	-	-	+	+	+	-	-	-	-	4
Fryer 2002	[16]	+	+	-	-	-	+	-	+	+	+	+	-	+	-	8
Gajdosik 2005	[5]	+	+	+	+	-	-	-	+	+	+	+	-	+	-	9
Gajdosik 2007	[18]	+	+	-	+	-	-	-	-	+	+	+	-	-	-	6
Grieve 2011	[2]	+	+	-	-	-	-	+	+	+	+	+	-	-	-	7
Johanson 2009	[3]	+	+	-	+	-	-	-	-	+	+	+	-	+	-	7
Kasser 2009	[19]	+	+	-	-	-	-	+	+	+	+	-	-	-	-	6
Knight 2001	[21]	+	+	-	+	-	-	+	+	+	+	+	-	-	-	8
Macklin 2012	[6]	+	-	-	-	-	-	-	+	+	+	+	-	-	-	5
McNair 1996	[22]	-	-	-	-	-	-	-	+	+	+	+	-	-	-	4
Peres 2002	[23]	+	+	-	-	-	-	-	-	+	+	+	-	+	-	6
Pratt 2003	[32]	-	+	-	-	-	-	-	+	+	+	+	-	+	-	6
Rees 2007	[25]	+	+	-	-	-	-	-	+	+	+	+	-	+	-	7
Samukawa 2011	[27]	-	-	-	-	-	-	-	+	+	+	+	-	+	-	5
Venturini 2007	[40]	+	-	-	-	-	-	-	-	+	+	+	-	+	-	5
Youdas 2003	[29]	+	+	-	+	-	-	-	+	+	+	+	+	+	-	9
Zakas 2006	[30]	-	-	-	-	-	-	-	+	+	+	+	-	+	-	5

1. Eligibility criteria were specified.

2. Subjects were randomly allocated to groups.

3. Allocation was concealed.

4. The groups were similar at baseline regarding the most important prognostic indicators.

5. There was blinding of all subjects.

6. There was blinding of all therapists who administered the therapy.

7. There was blinding of all assessors who measured at least one key outcome.

8. Measures of at least one key outcome were obtained from more than 85% of the subjects initially allocated to groups.

9. All subjects for whom outcome measures were available received the treatment or control condition as allocated.

10. The results of between group statistical comparisons are reported for at least one key outcome.

11. The study provides both point measures and measures of variability for at least one key outcome.

12. Was the sample size justified?

13. Were the outcome measures reliable?

14. Were the outcome measures valid?

The modified PEDro scale featured three additional assessment criteria taken from Law et al. [36]. The additional criteria assessed whether the sample size was justified and whether the outcome measures used were both reliable and valid. The assessment scale featured 14 criterion designed to assess the methodological quality of randomised and non-randomised trials. Trials were awarded a ‘yes’ or ‘no’ rating for each criteria and ‘yes’ responses were then summed to produce an overall quality score for each trial assessed. For consistency, any criteria not directly reported by the authors of each trial were considered to have been unfulfilled and subsequently were awarded a ‘no’ rating.

### Data extraction and analysis

Data extraction was performed by a single investigator (RY), who recorded details regarding study design, sample characteristics, outcome measures, interventions and follow-up periods. In order to calculate effect sizes, means and standard deviations (SD) were obtained wherever possible for each study group. In two studies [15,25] a smaller reported value for ankle ROM indicated greater dorsiflexion, so in these cases the means were subtracted from 90 degrees prior to analysis to allow for standardisation against other presented results. Standardised mean differences (SMDs) and 95% confidence intervals (CIs) were calculated, based on the difference between treatment and control groups at the longest period of follow-up. Where studies did not include a comparison group, SMDs were not calculated. SMDs were considered statistically significant if the 95% CI did not contain zero, and interpretation of the magnitude of SMDs was based on previous guidelines [37]: small effect ≥ 0.2, medium effect ≥ 0.5, large effect ≥ 0.8. Positive effect sizes indicated greater increases in ankle joint dorsiflexion in the treatment group compared to the control group. Where more than one intervention group was compared, intervention group A was designated to be the reference group for analysis. Random effects meta-analysis methods were used to pool data where study methods were considered to be homogenous. Sensitivity analyses were performed to investigate the influence of differing intervention or assessment techniques. Heterogeneity was quantified using Chi-squared tests (p < 0.10) and the I^2^ statistic described by Higgins et al. [38], which represents the percentage of total variation across studies due to heterogeneity. To investigate potential bias across studies included in the meta-analysis, effect sizes were plotted against study quality score and sample size, and symmetry of these plots was assessed visually. All statistical analyses were performed using Stata version 10 (StataCorp LP, College Station, TX).

## Results

### Included studies

**Figure 1:**
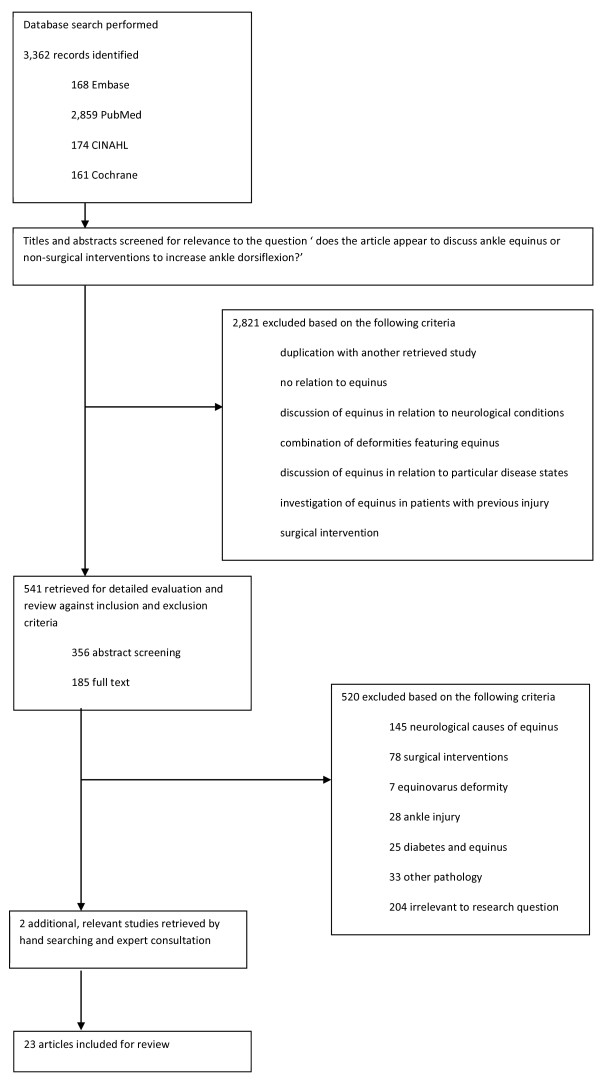
Selection process for study inclusion.

The search strategy returned 3,362 studies total from five databases (Figure 1). A further two potentially relevant studies were sourced through expert consultation. After initial screening to exclude irrelevant studies and to remove duplicates, 541 studies remained for detailed evaluation. The abstracts were read in order to select those that were directly relevant to this review. Three hundred and fifty-six studies were excluded based on abstract screening, and full text evaluations were performed on the remaining 185 studies. Twenty-three studies including a total of 734 participants satisfied the inclusion criteria for this review. Selected characteristics of included studies are presented in Additional file 1.

### Methodological quality

The 23 included studies received overall quality scores ranging from 1/14 to 11/14 on the modified PEDro scale. Results from quality assessment are presented in Table 1. Of the studies selected for inclusion, 13 featured a control group [2,3,5,12,16,18,19,21],[23,25,29,31,32,38]. Sixteen of the included studies randomly assigned participants to treatment groups [1-3,5,12,14-16,18,19,21,23],[25,29,31,32], nine studies featured blinding of the participants, assessors or therapists [1,2,12,13,16,19,21,31],[38] and 13 studies reported on the reliability of the measures used [1,3,5,12,14,16,23,25],[27,29-32]. No studies reported on the validity of measures employed.

### Effects of interventions

Eighteen studies investigated stretching interventions [1,3,5,6,12,14,15,18],[19,21-23,25,27,29-32] and six of the 18 studies investigated the effects of combining stretching with interventions such as ultrasound therapy or strengthening exercises [14,19,21-23,30]. Two studies investigated manipulation therapy [13,16], two studies investigated mobilisation therapy [38,39] and one investigated soleal trigger point therapy [2]. Four studies did not include a comparison group [6,13,27,39] and a further four studies [19,31,32,39] reported insufficient data for SMDs to be calculated. Data comparing the effectiveness of various interventions, including SMDs and 95% CIs, is presented in Table 2. Table 3 provides a synthesis of the body of evidence for each intervention.

**Table 2:** Comparison of conservative interventions for increasing ankle joint dorsiflexion range of motion

**Study ID**	**Intervention(s)**	**Sample size**	**Follow-up period**	**Measurement method**	SMD (95% CI)*
**STRETCHING**					
Bohannon 1994 [31]	A: Control	A: 18	Same day measures taken after 3 sets of stretching	Digital images	Insufficient data
	B: Stretch	B: 18			
Dinh 2011 [1]	A: WB stretch	A: 14	3 weeks	Goniometer (WB)	B vs A
	B: NWB stretch	B: 14			Left: -0.33 (−1.08 to 0.42)
					Right: 0.26 (−0.49 to 1)
				Goniometer (NWB)	B vs A
					Left: 0.16 (−0.88 to 0.9)
					Right: 0.18 (−0.56 to 0.93)
Christiansen 2008 [12]	A: Control	A: 20	8 weeks	Goniometer (NWB)	B vs A: 0.71 (0.07 to 1.35)
	B: Stretch	B: 20			
Etnyre 1986 [15]	A: Static stretch	A: 12	3 sessions	Goniometer (active assist)	B vs A: -0.04 (−0.85 to 0.76)
	B: Contract-relax PNF stretch	B: 12			
		C: 12			C vs A: 1.90 (0.92 to 2.88 )
	C: Contract-relax-agonist-contract PNF stretch				
Gajdosik 2005 [5]	A: Control	A: 9	8 weeks	Electro-goniometer	B vs A: 0.69 (−0.24 to 1.62)
	B: WB stretch	B: 10			
Gajdosik 2007 [18]	A: Control	A: 4	6 weeks	Electro-goniometer	B vs A: 0.91 (−0.44 to 2.25)
	B: WB stretch	B: 6			
Johanson 2009 [3]	A: Control	A: 8	3 weeks	Goniometer	B vs A
	B: WB stretch	B: 8			Left: 1.19 (0.11 to 2.26)
					Right: 0.55 (−0.45 to 1.55)
Kasser 2009 [19]	A: Control	A: 9	6 weeks	Universal goniometer	Insufficient Data
	B: WB stretch	B: 9			
Knight 2001 [21]	A: Control	A: 18	6 weeks	Goniometer (passive ROM)	B vs A: 0.71 (0.05 to 1.38)
	B: Static Stretch	B: 19			
				Goniometer (active ROM)	B vs A: 0.7 (0.03 to 1.36)
Peres 2002 [23]	A: Control	A: 8	3 weeks	Digital inclinometer	B vs A: 0.85 (−0.10 to 1.81)
	B: Stretch	B: 11			
Pratt 2003 [32]	A: Control	A: 12	3 days	Digital images	Insufficient data
	B: Stretch	B: 12			
Rees 2007 [25]	A: Control	A: 10	4 weeks	Goniometer	B vs A
	B: PNF stretch	B: 10			Left: 0.82 (−0.1 to 1.74)
					Right: 0.84 (−0.08 to 1.76)
Youdas 2003 [29]	A: Control	A: 24	6 weeks	Goniometer (active assist)	B vs A: 0.45 (−0.14 to 1.04)
	B: 30 sec stretch	B: 22			
	C: 1 minute stretch	C: 22			C vs A: 0.24 (−0.34 to 0.83)
	D: 2 minute stretch	D: 21			
					D vs A: 0.46 (−0.14 to 1.05)
**STRETCHING COMBINED WITH OTHER INTERVENTIONS**					
Draper 1998 [14]	A: Stretch	A: 20	10 sessions	Inclinometer	B vs A: 0 (−0.62 to 0.62)
	B: Ultrasound + Stretch	B: 20			
Kasser 2009 [19]	A: WB stretch	A: 9	6 weeks	Universal goniometer	Insufficient data
	C: Tibialis anterior strengthening	C: 9			
Knight 2001 [21]	A: Control	A: 18	6 weeks	Goniometer (passive ROM)	C vs A: 0.70 (0.04 to 1.37)
	C: Heel raise + static stretch	C: 19			
		D: 21			D vs A: 0.84 (0.18 to 1.50)
	D: Superficial moist heat + static stretch	E: 20			
					E vs A: 0.95 (0.27 to 1.62)
	E: Ultrasound + static stretch				
				Goniometer (active ROM)	C vs A: 0.77 (0.10 to 1.44)
					D vs A: 0.65 (0 to 1.30)
					E vs A: 0.91 (0.24 to 1.58)
McNair 1996 [22]	A: WB soleus stretch	A: 24	3 sessions	Electro-goniometer	B vs A: 0.05 (−0.52 to 0.62)
	B: Aerobic exercise	B: 24			
Peres 2002 [23]	A: Control	A: 8	3 weeks	Digital Inclinometer	C vs A: 1.12 (0.05 to 2.18)
	C: Stretch +	C: 8			
	Diathermy	D: 9			D vs A: 1.16 (0.12 to 2.20)
	D: Stretch + Diathermy + Ice				
Zakas 2006 [30]	A: Warm up	A:18	3 sessions	Flexometer	B vs A: 0.72 (0.04 to 1.39)
	B: Stretch	B: 18			
	C: Warm up + stretch	C: 18			C vs A: 0.87 (0.18 to 1.55)
**MANUAL THERAPY**					
Fryer 2002 [16]	A: Control	A: 41	Immediate	Dynamometer (NWB)	B vs A: 0 (−0.44 to 0.44)
	B: Manipulation	B: 40			
De Souza 2008 [39]	A: Control	A: 25	Immediate	Biplane goniometer	B vs A: 0.19 (−0.37 to 0.75)
	B: Mobilisation	B: 25			
**SOLEAL TRIGGER POINT THERAPY**					
Grieve 2011 [2]	A: Control	A: 10	Immediate	Goniometer (NWB assisted)	B vs A: 0.72 (−0.18 to 1.63)
	B: Soleal trigger point therapy	B: 10			

*Abbreviations:WB* weight bearing, *NWB* non-weight bearing.

*SMDs (95% CIs) were calculated between groups at the longest period of follow-up.

**Table 3:** Synthesis of evidence for stretching, mobilisation, manipulation and soleal trigger point therapy

**Factor**	**Stretching**	**Mobilisation**	**Manipulation**	**Soleal trigger point therapy**
**Total number of studies (k)**	k = 18	k = 2	k = 2	k = 1
**Study designs**	RCT: k = 11	Experimental: k = 2	RCT: k = 1	RCT: k = 1
	Experimental: k = 7		Experimental: k = 1	
**PEDro score (range and median score)**	Range: 4 to 11	Range: 5 to 8	Range: 4 to 8	Score = 7
	Median: 7.5	Median: 6.5	Median: 6	
**Consistency of findings**	Significant effect: k = 4	Non-significant effect: k = 1	Non-significant effect: k = 1	Non-significant effect: k = 1
	SMD range: 0.70 (0.04 to 1.37) [21] to 1.69 (0.53 to 2.85) [3]	SMD 0.19 (−0.37 to 0.75) [39]	SMD 0 (−0.44 to 0.44) [16]	SMD 0.72 (−0.18 to 1.63) [2]
	Non-significant effect: k = 5	Insufficient data: k = 1 [40]	Insufficient data: k = 1 [13]	
	SMD range: 0.36 (−0.44 to 1.17) [32] to 0.91 (−0.44 to 2.25) [18]			
	Insufficient data or no control group comparison:			
	k = 9 [1,6,14,15,19,22,27,30],[31]			

### Stretching

**Figure 2:**
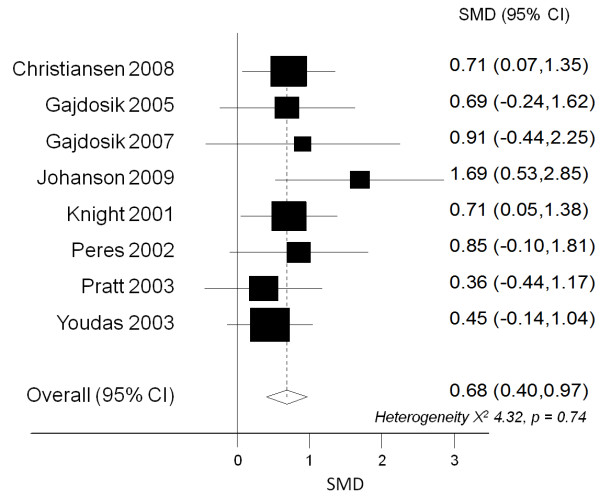
**Pooled effect size (SMD, 95% CI) for studies investigating stretching compared to control group.** Positive effect sizes indicate greater increases in ankle joint dorsiflexion in the treatment group compared to the control group.

Of the 18 studies that investigated stretching interventions, seven studies did not compare to a control group [1,6,14,15,22,27,30] and two studies presented insufficient data [19,31]. Another study investigating proprioceptive neuromuscular facilitation stretching was excluded from the meta-analysis due to differing stretching technique [25]. The results of eight remaining studies [3,5,12,18,21,23,29,32] investigating static stretching interventions were combined by meta-analysis methods. The combined effect size indicated that stretching had a statistically significant effect on increasing ankle joint dorsiflexion (SMD 0.68, CI: 0.40 to 0.97) (Figure 2). There was no statistically significant heterogeneity between pooled studies (Chi-squared 4.32, p = 0.74) (Figure 2). Visual inspection of funnel plots revealed that study quality score and sample size did not appear to bias the findings of studies included in the meta-analysis. Further analyses showed that removing studies from the meta-analysis that had used a non-weight bearing stretching technique, or used a different assessment technique (active versus passive ROM or knee flexed versus knee extended) did not substantially alter the findings of the meta-analysis.

One study demonstrated that proprioceptive neuromuscular facilitation stretching was associated with greater increases in ankle dorsiflexion ROM than static stretching (SMD 1.90, 95% CI: 0.92 to 2.88) [15]. Stretching in combination with warming up (SMD 0.87, 95% CI: 0.18 to 1.55) [30], superficial moist heat (SMD 0.84, 95% CI: 0.18 to 1.50) [21], diathermy (SMD 1.12, 95% CI: 0.05 to 2.18) [23], diathermy and ice (SMD 1.16, 95% CI: 0.12 to 2.20) [23] and heel raise exercises (SMD 0.70, 95% CI: 0.04 to 1.37) [21] were also found to be associated with greater increases in ankle dorsiflexion when compared to controls. In contrast, 12 studies showed no significant differences in ankle joint dorsiflexion between the stretching and comparison groups (SMDs: range −0.84 to 1.19) [1,5,6,14,18,19,22,25],[27,29,31,32].

Two studies investigated the use of ultrasound prior to stretching compared to stretching alone. One reported significantly greater overall gains in ankle joint dorsiflexion ROM compared to stretching alone (SMD 0.95, 95% CI: 0.27 to 1.58) [21]. The second study found that there was no statistically significant difference between these two interventions. (SMD 0.0, 95% CI: -0.62 to 0.62) [14].

### Manual therapy

Two studies eligible for inclusion in this review assessed the effect of manipulation therapy on ankle joint ROM. One study found that talocrural joint manipulation did not produce a statistically significant increase in dorsiflexion ROM in asymptomatic ankles when compared with a control group (SMD 0.0, 95% CI: -0.44 to 0.44) [16]. The second study reported insufficient data and was not included in the meta-analysis [13].

Two studies eligible for inclusion in this review assessed the effects of mobilisation therapy on ankle joint dorsiflexion ROM [39,40]. One study assessing the effects of mobilisation therapy did not report sufficient data to be included in the meta-analysis [40]. The second study found that talocrural joint mobilisation did not produce a statistically significant increase in dorsiflexion ROM when compared to a control group (SMD 0.19, 95% CI: -0.37 to 0.75) [39]. The second study assessing mobilisation therapy reported insufficient data and was not included in the meta-analysis [40].

### Soleus trigger point release

One included study investigated the effect of trigger point therapy on ankle joint dorsiflexion ROM and reported no statistically significant difference between the intervention and control groups (SMD 0.72, 95% CI: -0.18 to 1.63) [2].

## Discussion

This systematic review investigated the effects of conservative interventions on ankle joint dorsiflexion ROM in healthy individuals. Effect sizes calculated from individual studies revealed that stretching alone and the use of stretching in conjunction with ultrasound therapy, superficial moist heat, warm up and heel raise exercises were associated with statistically significant gains in ankle joint dorsiflexion ROM in the intervention groups compared to controls. There is currently insufficient evidence to suggest that soleal trigger point therapy, ankle joint mobilisation or manipulation are associated with statistically significant gains in ankle dorsiflexion ROM in healthy individuals.

Only five of the studies assessing the effects of stretching reported statistically significant gains in ankle ROM [3,12,21,30,31] however, the combined effect size from eight studies included in the meta-analysis showed a statistically significant positive effect of stretching compared to a control condition (pooled SMD 0.68, CI: 0.40 to 0.97) (Figure 2). Whilst the majority of studies investigating the efficacy of stretching programs reported non-significant results, it may be that this was due to small sample size or methodological weakness rather than genuine inefficacy of the intervention. Only three of the 18 studies investigating the efficacy of stretching on ankle dorsiflexion ROM reported conducting power calculations to determine necessary sample size [1,29,30].

There is some evidence to suggest that interventions such as stretching and the combined use of stretching with ultrasound, warm up, superficial moist heat and heel raise exercises are effective in the short term. As such, they may be considered suitable for use in patients where even short term increases in ankle dorsiflexion would clinically be considered beneficial. The minimal clinically important difference for ankle dorsiflexion ROM has not been established; however, studies included in this review reported differences of two [29] to eight [18] degrees between intervention and control groups at follow up.

With respect to study quality, of the 23 trials included for detailed analysis in this review, 13 included a control group [2,3,5,12,16,18,19,21],[25,29,31,32,39], whilst four studies did not include any comparison group [6,13,27,40]. Six compared interventions to each other without including a non-intervention control group [1,14,15,19,22,30]. Without comparing interventions to a control group, it cannot be known whether observed changes in ankle ROM may be attributed to real change or to the effects of individual variation at different points in time. Similarly, studies that compared two interventions to each other without comparing them to a control group, may only conclude that one intervention may be more beneficial than another. It can be noted that in this review, the ten non-controlled studies presented results which were similar to those reported in the 13 controlled studies.

All of the studies included for evaluation in this review assessed the effects of conservative interventions on ankle joint ROM in healthy individuals. The scope of this review did not include patients with neurologically-associated equinus deformity due to the current sound body of research pertaining to equinus deformity in individuals with neurological disease. A recent Cochrane review published by Rose et al. [34] investigated the efficacy of a range of conservative and surgical interventions in patients with neurologically-linked equinus deformity and reported that the use of night splints, prednisone and surgery were not associated with statistically significant increases in ankle joint dorsiflexion ROM in this sample population. The scope of this review also did not include individuals with active pathology such as acute metatarsalgia, plantar fasciopathy, Achilles tendinopathy, medial tibial traction periostitis or muscle strain. Consequently, future systematic reviews are warranted to determine the efficacy of conservative interventions in symptomatic populations such as these.

The majority of studies evaluated within this review reported on the short-term effects of conservative interventions on ankle joint dorsiflexion. The longest study period was eight weeks [5,6,12] and fewer than half of the included studies followed up their study participants for longer than four weeks. Due to the relative brevity of study periods, it is difficult to ascertain for how long conservative treatments may remain effective. As such, future research investigating the long term effects of conservative interventions to better inform clinical practice.

A review by Gatt et al. [41], investigated the reliability of a range of measurement techniques used to assess ankle dorsiflexion ROM. The review emphasized that the reliability and validity of goniometric measurements of ankle ROM have been shown to be poor and thus the use of goniometry in clinical trials calls into question the quality of the results obtained. The seven studies included in this review that presented reliability data for goniometry, reported ICCs between 0.80 and 1.00 [1,3,12,25,29,38,40]. It must be noted however, that none of the included studies reported on the validity of measures used.

In this review, change in ankle dorsiflexion ROM was used as the main outcome measure to assess the efficacy of conservative interventions. There are a number of potential limitations associated with using measures of ankle joint dorsiflexion in this way. Firstly, it has been suggested that conventional measures of ankle joint dorsiflexion actually assess combined dorsiflexion range of motion at the ankle and midtarsal joints rather than at the ankle alone [41,42]. Secondly, it is possible that ankle range of motion is not directly indicative of functional performance. Turner et al. [43] studied cohorts of diabetic patients and healthy adults in order to assess the correlation between passive and functional ROM measures at the ankle joint. It was reported that there was a lack of correlation between the two measures and thus concluded that passive ankle joint ROM may not accurately reflect functional limitations in joint mobility at the ankle joint.

Foot posture has a profound effect on the measurement of dorsiflexion range of motion at the ankle joint. A study by Tiberio et al. [44] concluded that measuring ankle joint dorsiflexion with the foot in a pronated position increases recorded ROM by up to 10 degrees when compared with measuring dorsiflexion in a subtalar joint neutral position. Of the 23 studies included in this review, seven studies reported that ankle joint range of motion was measured in subtalar joint neutral position to minimise the effects of foot posture on ROM [1,3,6,13,29,31,39]. The remaining 16 studies however, did not report any standardisation of foot posture during ROM measurements and consequently, there is question as to the validity and consistency of measures obtained.

Although clinicians often prescribe interventions to increase ankle joint dorsiflexion in patients with clinically diagnosed ankle equinus (less than 10 or 15 degrees of ankle dorsiflexion), seven of the studies included in this review sampled study participants who had an initial ankle dorsiflexion ROM greater than 10 to 15 degrees [14,22,27,30,32,38,40]. In light of this, there is some question as to the generalizability of the findings presented in this review to patients with equinus deformity.

Further research needs to be undertaken in the future to investigate the functional, as well as the statistical significance of conservative interventions. Rees et al. [25] reported that the increases in musculotendinous stiffness and ankle joint range of motion associated with PNF stretching would be beneficial for athletes participating in sports such as sprinting, and cycling. Gadjosik et al. [5] and Christiansen et al. [12] both reported that stretching of the ankle plantarflexors may be associated with significant functional improvements in older populations. None of these studies however report the magnitude of increase in ankle dorsiflexion necessary to produce clinically significant improvements in function. Until further high quality research is published therefore, clinicians must continue to incorporate their own clinical expertise and knowledge of individual patient needs with research-based evidence when developing treatment plans.

## Conclusion

There is some evidence to support the efficacy of stretching with or without the concurrent use of ultrasound, diathermy, diathermy and ice, heel raise exercises or warm up in increasing dorsiflexion range of motion at the ankle joint in healthy individuals. However, there is insufficient evidence to suggest that soleal trigger point therapy, ankle joint mobilisation or manipulation therapy are associated with statistically significant gains in ankle dorsiflexion range of motion. Current evidence is limited by inconsistent assessment methods and definitions of ankle equinus, as well as poor methodological rigor. Further research is required to investigate which conservative interventions are most effective for managing healthy individuals with ankle restricted ankle dorsiflexion range of motion.

## Abbreviations

ROM: Range of motion; SD: Standard deviation; SMD: Standardised mean difference; CI: Confidence interval.

## Competing interests

The authors declare that they have no competing interests.

## Authors’ contributions

RY performed the data searches, performed all quality assessments and data extractions and drafted the manuscript. SN was involved with study design, performed meta-analysis and data calculations and revised the manuscript. AW performed quality assessments and assisted with manuscript revisions. LR was involved with study conception and design, and assisted with manuscript revisions. RB was involved with the study conception and design and assisted with manuscript revisions. All authors read and approved the final manuscript.

## Supplementary Material

Additional file 1 Selected characteristics of included studies (23 studies). Click here for file
