# Supplementary material for: Interventions for increasing ankle joint dorsiflexion: a systematic review and meta-analysis
Source: J Foot Ankle Res. 2013 Nov 14;6:46. doi: 10.1186/1757-1146-6-46 (PMC4176290; doi:10.1186/1757-1146-6-46)
Supplement: Additional file 1 — Selected characteristics of included studies (23 studies). [file 1757-1146-6-46-S1.docx]

**Additional File 1 – Selected characteristics of included studies (23 studies)**

| **Study ID** | **Reference number** | **Study design** | **Sample** | **Intervention** |
| --- | --- | --- | --- | --- |
| Bohannon (1994) | [31] | Randomised control trial | 36 female volunteers randomly assigned to experimental or control groups. | Control group: nil intervention  Intervention group: 5 minutes of weight bearing triceps surae stretching |
| Christiansen (2008) | [12] | Randomised control trial. | 40 participants were recruited for the study and randomly assigned to an intervention. Only 37 completed the study. | Control group: nil intervention.  Intervention group: 8 week stretching program. Hip and ankle stretches were performed twice a day for 8 weeks. Each stretch was held for 45 seconds and repeated 3 times during each session. |
| Dananberg (2000) | [13] | Non-randomised experimental trial. | 22 patients between the ages of 20 and 69 with gastrocnemius equinus. Recruited from the Bedford Podiatry Group. | Manipulation of the proximal fibular head and talus. |
| De Souza (2008) | [38] | Non-randomised experimental trial. | 25 asymptomatic subjects between the ages of 18 and 35. | Maitland grades III and IV mobilisation of the right ankle. |
| Dinh (2010) | [1] | Experimental trial. | A convenience sample of 36 volunteers with less than 10 degrees of passive ankle dorsiflexion was recruited and 28 subjects completed the study. | Group 1: Non-weight bearing gastrocnemius stretching performed 5 times for 30 seconds each, twice a day for 21 days.  Group 2: Weight bearing gastrocnemius stretching performed 5 times for 30 seconds each, twice a day for 21 days. |
| Draper (1998) | [14] | Non-randomised experimental trial. | 40 healthy college students volunteered for participation. | Stretching alone: Static gastrocnemius and soleus stretches held for 20 seconds and repeated 4 times in total.  Stretching and ultrasound: 3MHz, 1.5 W/${cm}^{2}$ for 7 minutes applied to the musculotendinous junction of the right triceps surae. Immediately after ultrasound, subjects performed a gastrocnemius stretch for 20 seconds followed by a soleus stretch for 20 seconds. The stretching cycle was repeated three more times. |
| Etnyre (1986) | [15] | Non-randomised experimental trial. | 12 males aged between 21 and 33 were randomly assigned to a treatment order group. | Static stretching group: Passive soleal stretch held for 9 seconds.  Contract relax stretching group: passive lengthening of soleus, then isometric plantarflexion for 6 seconds, then a further 3 sec of passive soleal stretching.  Contract-relax-agonist-contract stretching group: Same as contract-relax except that pt assisted post-contraction dorsiflexion. |
| Fryer (2002) | [16] | Randomised control trial. | 41 healthy volunteers aged between 18 and 40. | Control group: nil intervention.  Treatment group: a single manipulation of the talocrural joint administered by an osteopath. High velocity, low amplitude thrust technique was used. |
| Gajdosik (2005) | [5] | Randomised control trial. | 19 older women with active ankle dorsiflexion range of motion <10 degrees were recruited from the community. | Control group: nil intervention.  Stretching group: Gastrocnemius stretching performed with Kin-com system for 10 repetitions of 15 seconds stretching. Repeated three times/week for 8 weeks. |
| Gajdosik (2007) | [18] | Randomised control trial. | 12 unconditioned women between 18 and 31 years volunteered for participation. | Control group: nil intervention.  Stretching group: ten static wall stretches for gastrocnemius held for 15 seconds each and repeated 5 times each week for 6 weeks. |
| Grieve (2011) | [2] | Randomised control trial. | 28 healthy physiotherapy and sports therapy students with unilateral restriction in active ankle dorsiflexion (<10 degrees) and at least one identifiable myofascial trigger point in the soleus muscle. | Control group: nil intervention.  Intervention group: Trigger point release performed on myofascial trigger points within the soleus. Barrier release technique was used. |
| Johanson (2009) | [3] | Randomised control trial. | 9 men and 7 women with less than 5 deg of passive ankle dorsiflexion with knee extended. | Experimental group: gastrocnemius stretches held for 30 seconds, repeated three times, twice each day for five weeks.  Control group: nil intervention. |

**Additional File 1 (continued)**

| **Study ID** | **Reference number** | **Study design** | **Sample** | **Intervention** |
| --- | --- | --- | --- | --- |
| Kasser (2009) | [19] | Randomised control trial. | 27 patients between 20 and 45 years with less than 20 degrees of active ankle dorsiflexion. | Control group: nil intervention.  Stretching group: gastrocnemius stretching performed with the ProStretch device. Stretches were held for 30 seconds and repeated three times on 5 days of each week for 6 weeks.  Strengthening group: tibialis anterior strengthening performed with a 10-lb ankle weight for three sets of 10 reps, 5 days each week for 6 weeks. |
| Knight (2001) | [21] | Randomised Control Trial. | 97 subjects from the local community volunteered for participation. | Group 1: control  Group 2: Static stretching  Group 3: Active heel raises prior to static stretching  Group 4: Superficial moist heat prior to stretching  Group 5: 7 minutes of continuous ultrasound prior to static stretching.  Static stretching involved 4 sets of 20 second gastrocnemius stretches performed three times each week for 6 weeks. |
| Macklin (2012) | [6] | Non-randomised experimental trial. | 13 runners with less than 6 deg of ankle joint dorsiflexion with the knee extended, but no bony block. | 4 minutes of calf stretching twice each day for 8 weeks using a Flexeramp device. |
| McNair (1996) | [22] | Experimental trial. | 24 recreational athletes with no musculoskeletal problems. | Stretching group: 5, 30 second static soleal stretches.  Aerobic exercise group: 10 min of treadmill running at 60% of maximum.  Combined protocol: aerobic exercise followed by stretches. |
| Pratt (2003) | [32] | Randomised Control Trial | 24 healthy volunteers randomly assigned to experimental or control groups. | Control group: nil intervention  Intervention group: 3 minutes of passive triceps surae stretching performed on three consecutive days with heels suspended from the edge of a platform. |
| Peres (2002) | [23] | Randomised Control Trial. | 44 healthy college students. | Control 1: nil intervention.  Control 2: nil intervention.  Treatment 1: Stretching.  Treatment 2: diathermy and stretching.  Treatment 3: diathermy, stretching and ice. |
| Rees (2007) | [25] | Randomised Control Trial. | 20 healthy, active women. | Proprioceptive neuromuscular facilitation stretching program. 3 sessions per week for 4 weeks of contract-relax agonist-contract PNF. |
| Samukawa (2011) | [27] | Non-randomised experimental trial. | 20 healthy male university students. | Dynamic plantarflexor muscle stretches. 5 repetitions of 30 seconds performed on right leg only. |
| Venturini (2007) | [39] | Experimental trial. | 35 healthy university students. | Maitland grade III anteroposterior joint mobilisation of the talus. |
| Youdas (2003) | [29] | Randomised control trial. | 101 volunteers. | Group 1: control  Group 2: 1 repetition of a static stretch of the right calf MTU once per day for 30 seconds  Group 3: 1 repetition of a static stretch of the right calf MTU once per day for 1 minute  Group 4: 1 repetition of a static stretch of the right calf MTU once per day for 2 minutes.  Stretches were repeated 5 days a week for 6 weeks. |
| Zakas (2006) | [30] | Non-randomised experimental trial. | 18 adolescent team soccer players. | Treatment control: active warm up (continuous jogging for 20 minutes)  Treatment 1: active warm up followed by stretching adductors, hamstrings, quadriceps, soleus, hip flexors and spinal extensors for 15 sec each repeated 3 times.  Treatment 2: Stretching alone |
